# Determinants of puerperal sepsis among postpartum women at a tertiary care hospital in Ethiopia: an unmatched case-control study

**DOI:** 10.1186/s40834-024-00283-x

**Published:** 2024-04-24

**Authors:** Keraj Seboka, Abenet Menene Gurara, Nardos Tilahun Bekele, Yohanes Abera Belachwe, Mihiret Shawel Getahun, Yohannes Mekuria Negussie

**Affiliations:** 1 Department of Nursing, Adama General Hospital and Medical College, Adama, Ethiopia; 2 Department of Nursing, Arsi University, Asella, Ethiopia; 3 Department of Public Health, Adama Hospital Medical College, Adama, Ethiopia; 4 Department of Public Health, Adama General Hospital and Medical College, Adama, Ethiopia; 5 Department of Medicine, Adama General Hospital and Medical College, Adama, Ethiopia

**Keywords:** Case-control, Determinants, Ethiopia, Puerperal sepsis

## Abstract

**Background:**

Puerperal sepsis, is a significant factor in maternal morbidity and mortality, especially in regions with lower income levels where maternal mortality rates are highest. However, it can be largely avoided if detected in time. Recognizing and dealing with the root causes early is essential in addressing this problem. Therefore, this study aimed to identify the determinants of puerperal sepsis among postpartum women at a tertiary care hospital in Ethiopia.

**Methods:**

An institutional-based unmatched case-control study was conducted among 266 postpartum women (88 cases and 178 controls) from October 1, 2023 to November 30, 2023. For each case, two controls were chosen using a systematic random sampling approach. Data were collected using an interviewer-administered, structured questionnaire and medical record review. The collected data were entered into Epi Info version 7.2 and analyzed using SPSS version 27. Binary logistic regression analysis was used to model the association between puerperal sepsis and independent variables. variables that had a crude association in the bivariable analysis (*p* < 0.25) were entered and analyzed by a multivariable binary logistic regression model to identify statistically significant factors. In the final model, Adjusted odds ratios with their 95% confidence intervals were calculated to determine the strength of the association. Statistical significance was declared at *p* < 0.05.

**Result:**

Rural residence (AOR = 6.9; 95% CI:2.77–17.10), having no formal education (AOR = 3.8; 95% CI: 2.55, 10.76), cesarean section delivery (AOR: 5.1; 95% CI: 1.30, 11.00) and complication during pregnancy (AOR: 4.6, 95% CI: 1.96, 11.10) were independent determinants of puerperal sepsis.

**Conclusion:**

Place of residence, maternal education level, mode of delivery, and complication during pregnancy were determinants of puerperal sepsis. It is crucial to implement education and awareness initiatives aimed at mothers, ensure universal access to healthcare services, advocate for evidence-based delivery protocols, and conduct comprehensive antenatal screenings.

## Introduction

Puerperal sepsis, an avoidable leading cause of maternal morbidity and mortality, is characterized by infections in the genital tract occurring during pregnancy, childbirth, or up to 42 days postpartum [1–3]. It stands as a preventable primary contributor to maternal morbidity and death [4]. According to the World Health Organization (WHO), approximately 11% of the estimated maternal deaths worldwide were attributed to sepsis [5–7]. In regions like sub-Saharan Africa and Southern Asia, a significant proportion of maternal fatalities is attributed to postpartum sepsis [8]. Recent global assessments indicate that obstetric infections rank as the third most prevalent cause of maternal death, with a prevalence mainly observed in low- and middle-income nations. Sub-Saharan Africa alone bears two-thirds of this burden, indicating a disproportionate impact on maternal mortality rates in the region [9–11].

Failure to detect and treat puerperal sepsis in its early stages can result in a cascade of health challenges, spanning both immediate and lasting impacts. These include prolonged hospital stays, pelvic inflammatory disease, disseminated intravascular coagulation, septicemia, wound dehiscence, pelvic abscess, peritonitis, septic pelvic thrombophlebitis, septic shock, organ injuries, infertility, and potential loss of life [12–14].

The primary challenge posed by puerperal sepsis stems not just from its complex array of causes but also from the numerous risk factors that increase the likelihood of a mother being susceptible to it [15]. Factors such as pre-existing maternal illness, place of residence, length of labor, prolonged rupture of membranes, parity, multiple vaginal examinations, manual placental removal, and method of delivery were notable contributors to puerperal sepsis [3, 16–20].

Despite Ethiopia’s efforts to improve healthcare access and emergency obstetric care, as well as implementing interventions for maternal health services to reduce maternal mortality, many mothers still die [21]. In 2000, the maternal mortality ratio was 1030 per 100,000 live births, decreasing to 412 per 100,000 live births according to the 2016 Ethiopia Demographic and Health Survey [22, 23]. However, there remains inadequate evidence, particularly within our study locale, to elucidate the determinants of puerperal sepsis, with contributing factors showing inconsistency across various studies. Acknowledging the mounting global burden of puerperal sepsis and its ramifications, our study aims to fill a significant gap in the current understanding of the determinants of puerperal sepsis within our study area. Therefore, this study aimed to identify the determinants of puerperal sepsis among postpartum women at a tertiary care hospital in Ethiopia. Comprehending the determinants underlying puerperal sepsis is paramount in crafting comprehensive prevention and intervention initiatives geared toward mitigating these factors. Such efforts are pivotal in driving down maternal morbidity and mortality rates linked to this condition. As a result, the findings of this study will enrich the existing literature, assisting in pinpointing factors associated with puerperal sepsis. This may offer avenues for treatment and, consequently, enhance the overall quality of life.

## Methods

### Study design, area, and period

An institutional-based unmatched case-control study design was utilized at Asella Teaching and Referral Hospital between October 1, 2023, to November 30, 2023. The hospital is situated in Asella town, approximately 175 km south-central from Addis Ababa, the capital of Ethiopia. Operating as a government hospital, it serves the community by offering both patient care services and educational programs. The institution provides a variety of healthcare services, catering to an estimated population of 3.5 million from 28 districts and 2 town administrations. On average, there were 5230 deliveries annually across all types [24].

### Study population, and eligibility criteria

All postpartum women receiving postnatal care at the hospital were considered the source population, while all postpartum women receiving postnatal care during the study period were considered the study population. The study units were individual postpartum women, who were selected as either cases or controls. Those who were seriously ill, unable to communicate, or experiencing mental health issues during the data collection period were excluded from the study.

#### Cases

Postpartum mothers receiving postnatal care at the hospital during the study period and diagnosed with puerperal sepsis.

Controls: Postpartum mothers receiving postnatal care at the hospital during the study period were confirmed to be free of puerperal sepsis.

### Sample size determination, and sampling procedure

The sample size was determined using Epi Info version 7.2 statistical software, assuming 80% power, a 95% confidence interval (CI), a 1:2 case-to-control ratio, and a 10% contingency for nonresponse. Different factors significantly associated with the outcome variable were taken into consideration, and the largest sample size was selected (Table 1).

**Table 1:** Sample size determination using Epi Info 7.0 StatCalc program

Variables	Percentage of controls exposed to the risk factor	Adjusted odds ratio	Sample size	Total sample size	Final sample size after 10% contingency	Reference
Residence	20.3%	3.0	Cases = 53 Controls = 105	158	176	Sahle et al., 2023 [17]
Number of vaginal examinations	12.4%	4.53	Cases = 35 Controls = 69	104	116	Tesfaye et al., 2023 [18]
Duration of rupture of membrane	4.4%	4.1	Cases = 88 Controls = 175	263	292	Sahle et al., 2023 [17]
Mode of delivery	20.7%	3.85	Cases = 30 Controls = 90	120	133	Demisse et al.,2019 [16]

Thus, this maximum sample size was derived from the exposure variable, the Duration of rupture of the membrane which gave the sample size of 263 (88 cases and 175 controls). After adding 10% contingency the final calculated sample size for this study becomes 292 (98 cases and 194 controls).

Postpartum mothers diagnosed with puerperal sepsis during the data collection period were consecutively selected as cases. This approach was adopted due to the rarity of the cases, and all available cases were included to meet the required sample size. Two controls for each case were chosen using a systematic random sampling technique. The sampling interval for selecting controls was calculated by dividing the average total number of deliveries over the previous two years for the study period by the total number of controls.

### Data collection procedure and quality control

Data was collected through a pretested, structured, interviewer-administered questionnaire and by reviewing medical records. The questionnaires were adapted from relevant literature with necessary modifications to suit the study’s context [3, 14–20]. A data abstraction format was used to extract essential information from patients’ records. Three trained midwives gathered data under the supervision of two general practitioners. The questionnaire was prepared in English and translated into Amharic and Afan Oromo, local languages, ensuring consistency through back-translation. Content validity was evaluated through consultation with a panel of experts. The internal consistency of the instruments was assessed using Cronbach’s alpha, the results revealing it was good. Additionally, the reliability of the instruments was confirmed through pre-testing. A pre-test on 5% of the total sample was at Adama Hospital Medical College one week before the actual data collection to refine the data abstraction format and questionnaire. Two days of training were provided to data collectors and supervisors, covering procedures and objectives. Continuous supervision and regular meetings were held among the team to ensure data collection quality and consistency.

### Data processing and statistical analysis

After coding and inputting data into Epi-Info version 7.2, the data were exported to the Statistical Package for Social Sciences (SPSS) Version 27 for cleaning and analysis. Descriptive statistics were employed to present key characteristics of the participants. Binary logistic regression analysis was used to model the association between the outcome and independent variables. In the bivariable logistic regression model, a significance level of < 0.25 was set as a threshold to select variables for multivariable logistic regression analysis, aiming to control confounding effects. The model was built using the standard approach. Hosmer and Lemeshow’s goodness-of-fit test assessed the model’s fitness, and the result showed a *p*-value of 0.356, indicating a good fit. The variance inflation factor was used to explore multicollinearity among explanatory variables, revealing values within the range of 1.001 to 1.038, indicating the absence of multicollinearity. Adjusted odds ratios (AOR) with a 95% confidence interval (CI) were employed in the final model to estimate the strength of the association. In the final model, variables with a *p*-value < 0.05 were considered statistically significant.

## Results

### Socio-demographic characteristics

A total of 266 postpartum mothers, comprising 88 cases and 178 controls, participated in the study, resulting in a response rate of 91.1%. The mean age of the participants was 26.85 (SD: ±5.45) for cases and 28.17 (SD: ±5.5) for controls. The majority of cases 73(83%) were rural residents while most controls 110(61.8%) were urban residents. Among the cases, 62(70.4%) were married, and for controls, the percentage was 121(68%). Additionally, 28(31.8%) of cases and 56(31.5%) of controls had attended primary school (Table 2).

**Table 2:** Sociodemographic characteristics of postpartum women at Asella teaching and referral hospital,2023

Variables	Cases (%)	Control (%)
**Age**		
15–19	6(6.8%)	11(6.2%)
20–24	14(15.9%)	46(25.8%)
25–30	33(37.5%)	91(51.1%)
30–34	20(22.7%)	8(4.5%)
> 35	15(17.1%)	22(12.4%)
**Residence**		
Urban	15(17.0%)	110(61.8%)
Rural	73(83.0%)	68(38.2%)
**Marital status**		
Married	62(70.4%)	121(68.0%)
Single	15(17.1%)	40(22.5%)
Others*	11(12.5%)	17(9.5%)
**Educational status of the mother**		
No formal education	29(33.0%)	23(12.9%)
Primary	28(31.8%)	56(31.5%)
Secondary	16(18.2%)	53(29.8%)
College and above	15(17.0%)	46(25.8%)
**Educational status of husband**		
No formal education	14(15.9%)	26(14.6%)
Primary	27(30.7%)	62(34.8%)
Secondary	29(33.0%)	43(24.2%)
College and above	18(20.4%)	46(25.8%)
**Moher’s occupation**		
Government employee	7(8.0%)	13(7.3%)
Merchant	8(9.1%)	9(5.0%)
Housewife	69(78.4%)	140(78.7%)
Others**	4(4.5%)	16(9.0%)

**Notes**: *Divorced, widowed, **Daily labor, and non-governmental organization employee

### Obstetric related characteristics

In this study, 39 (44.3%) of the cases and 64 (36.0%) of the controls were multiparous. More than three-fifths (69.3%) of the cases and 60.1% of the controls gave birth through spontaneous vaginal delivery. Among the cases, 76 (86.4%), while 171 (96.1%) among controls gave birth at health institutions. From the participants, 71 (80.7%) of the cases and 152 (85.4%) of the controls had ANC follow-up. Approximately one-fifth (18.6%) of the cases and 11.8% of the controls were referred from one health institution to another during labor (Table 3).

**Table 3:** Obstetrics-related factors of postpartum women at Asella teaching and referral hospital, 2023

Variables	Cases (%)	Control (%)
**Parity**		
1	22(25.0%)	54(30.3%)
2–3	39(44.3%)	64(36.0%)
≥ 4	27(30.7%)	60(33.7%)
**Duration of rupture of membrane (hour)**		
< 24	85(96.6%)	169(94.9%)
≥ 24	3(3.4%)	9(5.1%)
**Duration of labor (hour)**		
< 6	26(29.5%)	26(14.6%)
6–12	12(13.6%)	66(37.1%)
12–24	49(55.7%)	75(42.1%)
≥ 24	1(1.2%)	11(6.2%)
**Mode of delivery**		
SVD	61(69.3%)	107(60.1%)
Instrumental delivery	19(21.6%)	57(32.0%)
Cesarean delivery	8(9.1%)	14(7.9%)
**Placental delivery**		
CCT	15(17.1%)	150(84.3%)
Manual removal	73(82.9%)	28(15.7%)
**Number of vaginal examinations**		
< 5	37(42.0%)	41(23.0%)
≥ 5	51(58.0%)	137(77.0%)
**Place of delivery**		
Home	12(13.6%)	7(3.9%)
Health institution	76(86.4%)	171(96.1%)
**ANC follow up**		
Yes	71(80.7%)	152(85.4%)
No	17(19.3%)	26(14.6%)
**Number of ANC visits (** ***n*** ** = 71)**		
≤ 2 times	20(28.2%)	62(40.8%)
≥ 3 times	51(71.8%)	90(59.2%)
**Complication during pregnancy**		
Yes	64 (72.7%)	56(31.5%)
No	24(27.3%)	122(68.5%)
**Referral status**		
Referred in	51(58.0%)	104(58.4%)
Not referred in	37(42.0%)	74(41.6%)

**Abbreviations**: ANC: antenatal care, SVD: spontaneous vaginal delivery, CCT: controlled cord traction.

### Maternal health-related characteristics

From the postpartum women included in this study, 17 (19.3%) of the cases and 57 (32%) of the controls had information about puerperal sepsis. Additionally, 6 (6.8%) of the cases and 21 (11.8%) of the controls had urinary tract infections (UTIs) during their last pregnancy. Furthermore, 3 (3.4%) of the cases and 2 (1.1%) of the controls had HIV/AIDS (Table 3).

### Determinants of puerperal sepsis

After conducting bivariable binary logistic regression analysis, factors such as place of residence, educational status of the mother, mode of delivery, information about puerperal sepsis, place of delivery, and complications during pregnancy exhibited statistically significant associations. After adjustment for potential confounding factors via multivariable binary logistic regression analysis, place of residence, the educational status of the mother, mode of delivery, and complications during pregnancy emerged as independent determinants of puerperal sepsis at a *p*-value < 0.05.

Hence, the odds of puerperal sepsis were 6.9 times greater among postpartum women residing in rural areas compared to those residing in urban areas (AOR = 6.9; 95% CI: 2.77–17.10). Compared to mothers with a college education or higher, mothers with no formal education had 3.8 times higher odds of puerperal sepsis (AOR = 3.8; 95% CI: 2.55–10.76). Postpartum women who gave birth by cesarean delivery had 5 times increased odds of puerperal sepsis compared to mothers who delivered by spontaneous vaginal delivery (AOR: 5.1; 95% CI: 1.30–11.00). Moreover, the odds of developing puerperal sepsis were 4.6 times higher in mothers who had complications during their pregnancy compared to their counterparts (AOR: 4.6, 95% CI: 1.96–11.10) (Table 4).

**Table 4:** Maternal health-related characteristics of postpartum women at Asella teaching and referral hospital,2023

Variables	Cases (%)	Control (%)
**Information about puerperal sepsis**		
Yes	17(19.3%)	57(32.0%)
No	71(80.7%)	121(68.0%)
**STI during last pregnancy**		
Yes	12(13.6%)	7(3.9%)
No	76(86.4%)	171(96.1%)
**UTI during last pregnancy**		
Yes	6(6.8%)	21(11.8%)
No	82(93.2%)	157(88.2%)
**Hypertension**		
Yes	11(12.5%)	29(16.3%)
No	77(87.5%)	149(83.7%)
**Diabetes mellitus**		
Yes	8(9.1%)	21(11.8%)
No	71(90.1%)	157(88.2%)
**HIV/AIDS**		
Yes	3(3.4%)	2(1.1%)
No	85(96.6%)	176(98.9%)
**Vaginal douching**		
Yes	13(14.8%)	19(10.7%)
No	75(85.2%)	159(89.3%)

**Abbreviations**: STI: sexually transmitted infections, UTI: urinary tract infection

**Table 5:** Determinants of puerperal sepsis among postpartum women at Asella Teaching and referral hospital,2023

Variables	Cases (%)	Control (%)	COR (95% CI)	AOR (95% CI)
**Residence**				
Rural	73(83.0%)	68(38.2%)	7.9 (4.10, 14.82) *	6.9 (2.77, 17.10) **
Urban	15(17.0%)	110(61.8%)	1	1
**Educational level**				
No formal education	29(33.0%)	23(12.9%)	3.9 (1.74, 8.60) *	3.8 (2.55, 10.76) **
Primary	28(31.8%)	56(31.5%)	1.5 (0.73, 3.20)	1.7 (0.59, 5.10)
Secondary	16(18.2%)	53(29.8%)	0.9 (0.40, 2.10)	1.09 (0.61. 5.60)
College and above	15(17.0%)	46(25.8%)	1	1
**Mode of delivery**				
SVD	61(69.3%)	107(60.1%)	1	1
Instrumental delivery	19(21.6%)	57(32.0%)	0.9 (0.32, 1.10)	1.02 (0.40, 3.37)
Cesarean delivery	8(9.1%)	14(7.9%)	1.1 (0.40, 2.50) *	5.1 (1.30, 11.00) **
**Information about puerperal sepsis**				
Yes	17(19.3%)	57(32.0%)	0.5 (0.30, 0.94) *	1.4 (0.51, 3.70)
No	71(80.7%)	121(68.0%)	1	1
**Place of delivery**				
Home	12(13.6%)	7(3.9%)	3.9 (1.50, 10.20) *	1.6 (0.20, 15.60)
Facility	76(86.4%)	171(96.1%)	1	1
**Complication during pregnancy**				
Yes	64 (72.7%)	56(31.3%)	5.8 (3.30, 10.23)	4.6 (1.96, 11.10) **
No	24(27.3%)	122(68.5%)	1	1

**Notes**: *Significant at *p*-value < 0.25 in unadjusted logistic regression analysis, **significant at *p* < 0.05 in adjusted logistic regression analysis, 1 = Reference.

**Abbreviations**: AOR: adjusted odds ratio; CI: confidence interval; COR: crude odds ratio; SVD: spontaneous vaginal delivery.

## Discussion

This study aimed to assess the determinants of puerperal sepsis among postpartum women at Asella Teaching and Referral Hospital, Ethiopia. Place of residence, educational status of the mother, mode of delivery, and complications during pregnancy were identified as determinant factors for the occurrence of puerperal sepsis.

In this study, mothers living in rural areas had higher odds of developing puerperal sepsis than urban residents. This finding is congruent with studies conducted in Tigray, Ethiopia [17], and Oromia, Ethiopia [16]. The possible reason may be due to the lack of nearby healthcare facilities and inadequate sanitation and hygiene practices, as well as low health-seeking behaviors among mothers residing in rural areas. This may result in delayed scheduling of ANC appointments, home births in unsanitary conditions, and postponed seeking of medical assistance, all of which aggravate the risk of puerperal sepsis [25, 26].

The odds of developing puerperal sepsis were greater among mothers with no formal education compared to those who had a college education or higher. The finding is in line with studies conducted in the Oromia region, Ethiopia [16], Northwest, Ethiopia [19], Pakistan [27], and California, USA [28]. Education frequently correlates with health literacy, impacting mothers’ abilities to identify signs of infection, promptly seek medical care, and follow preventive measures. Thus, uneducated women may face these challenges, heightening their susceptibility to sepsis. Furthermore, education influences health behaviors such as hygiene, diet, and healthcare use. Mothers with lower education levels may be less likely to adopt healthy practices and follow prenatal and postnatal care recommendations, which can increase their risk of infections and complications [29, 30].

The current study revealed that the odds of developing puerperal sepsis were 5 folds greater among postpartum women who gave birth by cesarean delivery compared to those who delivered by spontaneous vaginal delivery. This result is comparable with different studies from the Oromia region, Ethiopia [16], Tigray region, Ethiopia [17], Hawassa City, Ethiopia [18], Northwest, Ethiopia [19], Gondar University referral hospital, Ethiopia [20], a systematic review and meta-analysis of seven studies in Ethiopia [3]. Studies done in Nigeria [31], Kenya [32],Tanzania [33], India [34], Germany [35], Scotland [36], Ireland [37] and systematic review and meta-analysis of six studies [38] also indicated a comparable finding. The possible reason for the elevated risk of developing puerperal sepsis in women who have given birth by cesarean delivery might be due to a combination of factors, including inadequate infection prevention during surgery, excessive blood loss, subpar sterile techniques in operating rooms, and insufficient post-operative hygiene. Additionally, complications such as prolonged labor, extended rupture of membranes, and hemorrhage, which necessitate cesarean section surgery, further elevate the risk of infection [39, 40].

Moreover, consistent with a study conducted in Northwest Ethiopia [19], the findings of this study revealed that the odds of developing puerperal sepsis were higher in mothers who had complications during their pregnancy compared to their counterparts. The justification for the increased risk of puerperal sepsis in women with complications during pregnancy lies in the various physiological and situational factors that make them more susceptible to bacterial infections. Complications such as tissue damage, invasive procedures, weakened immune systems, prolonged hospitalization, and underlying medical conditions create an environment conducive to bacterial growth and transmission.

### Limitations of the study

limitations of the study include the inherent constraints of the case-control design, which preclude establishing causal relationships, and potential recall bias in self-reported data.

## Conclusion

Place of residence, maternal education level, mode of delivery, and complication during pregnancy were all found to be independent determinants of puerperal sepsis. Different stakeholders should prioritize improving access to healthcare services, particularly in rural and underserved areas, and promote evidence-based delivery practices while implementing robust antenatal care programs to effectively monitor and manage pregnancy complications. Strengthening maternal education initiatives is also crucial to empowering pregnant women with knowledge, ultimately reducing the development of puerperal sepsis and improving maternal health outcomes.

## Data Availability

All data and materials are available from the corresponding author without undue reservation.

## Abbreviations

AOR: Adjusted Odds Ratio
ANC: Antenatal Care
CCT: Controlled Cord Traction
CI: Confidence Intervals
COR: Crude Odds Ratio
OR: Odds Ratio
SPSS: Statistical Package for Social Sciences
STI: Sexually Transmitted Infections
SVD: Spontaneous Vaginal Delivery
UTI: Urinary Tract Infection
